# The Bioequivalence of Emulsified Isoflurane With a New Formulation of Emulsion: A Single-Center, Single-Dose, Double-Blinded, Randomized, Two-Period Crossover Study

**DOI:** 10.3389/fphar.2021.626307

**Published:** 2021-03-10

**Authors:** Hui Yang, Qinqin Yin, Luying Huang, Min Zhang, Xinxin Zhang, Qirong Sun, Xuewei Liu, Qi Wang, Xi Yang, Lingcan Tan, Mao Ye, Jin Liu

**Affiliations:** ^1^ Department of Anesthesiology, West China Hospital, Sichuan University, Chengdu, China; ^2^ Department of Clinical Research Management, West China Hospital, Sichuan University, Chengdu, China; ^3^ Clinical Research Center, Yichang Humanwell Pharmaceutical CO., LTD, Yichang, China

**Keywords:** emulsified isoflurane, C_max_, AUC_0-t_, AUC_0-∞_, bioequivalence

## Abstract

**Background:** Emulsified isoflurane is a novel intravenous general anesthetic obtained by encapsulating isoflurane molecules into emulsion. The formulation of emulsion has been improved according to the latest regulations of the China Food and Drug Administration. This study was designed to compare the bioequivalence of the new and previous formulation emulsion of isoflurane.

**Methods:** In a single-center, single-dose, double-blinded, randomized, two-period crossover study, healthy volunteers received intravenous injection of 30 mg/kg of isoflurane with either previous formulation of emulsion isoflurane (PFEI) or new formulation of emulsion isoflurane (NFEI). Arterial and venous blood samples were obtained for geometric mean test/reference ratios of C_max_, AUC_0-t_, and AUC_0-∞,_ as well as their 90% confidence interval (CI90) as the primary outcome. The secondary outcomes were safety measurements such as vital signs, 12-lead electrocardiography, adverse effects, and laboratory tests; and anesthesia efficacy was assessed by Modified Observer’s Assessment of Alertness/Sedation (MOAA/S) score, bispectral index (BIS), and loss/recovery of eyelash reflex.

**Results:** 24 subjects were eligible, of which 21 completed the whole experiment (NFEI n = 21, PFEI n = 23). Arterial geometric mean test/reference ratios of C_max_, AUC_0-t_, and AUC_0-∞_ were 104.50% (CI90 92.81%–117.65%), 108.23% (94.51%–123.96%), and 106.53% (93.94%∼120.80%), respectively. The most commonly seen adverse effects for NFEI and PFEI were injection pain (38.1% vs. 34.8%), hypotension (19.0% vs. 13.0%), apnea (14.3% vs. 17.4%), and upper airway obstruction (14.3% vs. 13.0%). No severe adverse effect was observed. The effectiveness of general anesthesia was similar between the two formulations.

**Conclusion:** The CI90 of C_max_, AUC_0-t_, AUC_0-∞_, NFEI, and PFEI were within the range of 80%–125%, suggesting bioequivalence between NFEI and PFEI. The safety and anesthesia effectiveness were also similar.

## Introduction

Isoflurane is one of the most widely used volatile anesthetics. The application of isoflurane, however, requires special devices and causes environmental issues. Emulsified isoflurane (EI) is isoflurane emulsified in Intralipid^®^.

EI has several advantages over inhaled isoflurane. First, EI is an intravenous anesthetic that can be easily injected into veins (Fan et al., 2014; Diao et al., 2016) instead of requiring unique device, saving medical resources and reducing air pollution in the operating room. Second, EI does not need the lung uptake to take effects; it also avoids the dilution of the drug resulting from the respiratory circuit and functional alveolar residue capacity; therefore, induction is faster (Yang et al., 2008). Third, EI is eliminated mostly *via* expiration; anesthesia level can be easily controlled by injection speed and ventilation adjustment in the stage of anesthesia maintenance and emergence (Huang et al., 2010; Zhou, et al., 2011b; Huang et al., 2016; Wang et al., 2016; Yang et al., 2020; Zhao et al., 2020). Fourth, EI is more potent than isoflurane. EI has greater C_max_ and AUC (Yang et al., 2013). At some doses, the exposure of EI was greater than that of isoflurane (Huang et al., 2016; Wang et al., 2016; Yang et al., 2020; Zhao et al., 2020). In general, EI combines the advantages of both volatile and intravenous anesthetics (Huang et al., 2014), making it valuable in the clinical application (Zhou and Liu 2012). Several clinical studies have proved that EI is capable of rapid-onset, short-lasting general anesthesia in humans (Huang et al., 2014) and animals (Zhou et al., 2006; Yang et al., 2013; Fan et al., 2014; Diao et al., 2016).

To convert isoflurane into EI, the key step is to encapsulate isoflurane molecules into the emulsion. In the early stage of EI trials, isoflurane was simply stirred in 30% emulsion with shearing and homogenization process, which resulted in 350 nm particles. These particles are composed of isoflurane–soybean oil core and lecithin shell; during vibration and heat sterilization process, the particles are polymerized and eventually break down. Some broken particles lose the lecithin shell, merge into granules >5 μm, block pulmonary capillaries (diameter 7–9 μm), and result in cough immediately after drug application in some subjects (Zhou et al.,2011a; Xu et al., 2013; Zhang et al., 2014).

According to the latest regulations from the China Food and Drug Administration, and in order to address the large granules issue described above, a new formulation of the emulsion was developed. The concentration of lecithin was increased, and the soybean oil reduced. This change in emulsion formulation will reduce the size of particles and increase the stability of EI. As a result, large granules <0.05% were obtained. The new formulation of emulsified isoflurane (NFEI) should have the same bioequivalence as the previous formulation of emulsified isoflurane (PFEI) since the isoflurane–soybean oil core remains. Moreover, deduced from chemo-physical properties, NPEI may have the same efficacy in general anesthesia, or smoother induction, for example, less incidence of cough.

Healthy volunteers were enrolled to testify the bioequivalence, anesthesia efficacy, and safety of NFEI and PFEI (Xu et al., 2013). It is a hypothesis that NFEI has the same bioequivalence, anesthesia efficacy, and safety as those of PFEI.

## Methods

### Study Design and Approvals

This is a single-center, single-dose, double-blinded, randomized, two-period crossover study (ChiCTR1900025947), approved by the Chinese Food and Drug Administration of China (CYHB1803134) and West China Ethics Committee. Each healthy volunteer provided written informed content.

### Subjects Eligibility

Individuals were eligible if they are 18–45 years old, with body mass ≥45 kg for female and ≥ 50 kg for male and BMI of 19–26 kg/m^2^. Exclusion criteria included abnormal medical history/physical examination/vital signs/electrocardiogram (ECG)/laboratory test results, which were considered as clinically important; abuse of substances (except alcohol) that would affect the process of drug absorption, distribution, metabolism, or excretion; being positive for HIV, type B/C hepatic virus, or syphilis; allergy history or allergic to milk, pollen, or any drug gradient used in this study; alcohol addiction, or current alcohol consumption exceeding 21 (for male) or 14 (for female) units per week; for females, positive pregnant test or being in lactation period; tobacco consumption >5 cigarettes per day in the past 3 months before study entry; abnormal cognitive function test results; difficult airways (e.g., thyroid-mental distance ≤ 4 cm or Mallampati scores ≥3); previously participated in other clinical trials within three months; had donated blood within three months; and use of nicotine 48 h before study entry till the end of study.

### Study Protocol

Subjects were enrolled and randomized to receive intravenous 30 mg/kg of either NFEI or PFEI (1:1) *via* infusion pumps within 2 min (the infusion speed varied between subjects due to weight difference). After a washout of three days, these subjects had an intravenous injection of the other drug. Subjects received two-week fat-limited and no-alcohol/caffeine diet. All the volunteers fasted for 10 h before the study started. Before the test drug injection, all subjects inhaled oxygen (10 L/min) *via* face masks, with standard anesthesia monitored (T8, Mindray Medical International Ltd., Shenzhen, China) including 12-lead electrocardiogram (ECG), noninvasive blood pressure, pulse oximetry, respiratory rate, body surface temperature, bispectral index (BIS), and end-tidal carbon dioxide until subjects fully recovered. Vital signs were recorded continuously by the automatic monitor and manually at predefined time points: before drug application (as baseline), every minute for the first 15 min after dose, and every 5 min from 15 to 60 min after dose, respectively. One arm had venous and arterial catheterization before the experiment, to facilitate intravenous drug injection and arterial blood collection. Continuous noninvasive blood pressure monitoring and venous blood sample collection were performed in the other arm. Arterial blood and venous blood (4 ml, heparin treated) were simultaneously collected 10 min before drug injection and 1, 2, 2.5, 3, 4, 5, 7, 10, 20, 40, 60, and 70 min after intravenous drug administration. Baselines were recorded for vital signs (before and after oxygenation), consciousness state (Modified Observer’s Assessment of Alertness/Sedation, MOAA/S), and BIS readings before drug injection. This study was conducted in the Good Clinical Practice Center of West China Hospital of Sichuan University.

### Geometric Mean Reference Ratios of Pharmacokinetic Parameters

The primary outcomes for this study were geometric mean test/reference ratios of C_max_, AUC_0-t_, and AUC_0-∞_, as well as their 90% confidence interval (CI90). The geometric mean test/reference ratios were used to determine the bioequivalence of PFEI and NFEI (Bienvenu et al., 2017; Morton et al., 2018; Davanco et al., 2019). Other pharmacokinetic parameters were also investigated, including peak concentration (C_max_); area under the plasma concentration–time curve (AUC_0-t_); area under the concentration–time curve from time zero to infinity (AUC_0-∞_); time to plasma concentration peak (t_max_); elimination rate constant (λ_z_); half-life in the terminal elimination phase (t_1/2z_); the percentage of the area under the curve that has been derived after extrapolation (AUC_%Extrap_), calculated as [(AUC_0-∞_-AUC_0-t_)/AUC_0-∞_]×100%); mean residence time (MRT); plasma apparent clearance (CL_z_); and the apparent volume of distribution at the terminal elimination phase (V_z_).

### Testing of Plasma Concentration of Isoflurane

Blood samples were rapidly distributed into three headspace autosampler vials and stored in 2–8°C. The plasma concentration of isoflurane was tested by the two-stage headspace equilibrium gas chromatography method. Briefly, the blood sample at each test point was split into three parts: one for testing plasma concentration of isoflurane, one for incurred sample reanalysis (ISR), and one saved as backup. Plasma concentration of isoflurane was tested with headspace gas chromatography as previously reported (Yang et al., 2013). Isoflurane standard was used as an external standard. The determination range of isoflurane was 2.31 μg/ml to 500.00 μg/ml. The biological matrix was the whole blood sample. Agilent gas chromatograph 6890 N (instrument number: 20062567) with Agilent g1888 automatic sampler was used to detect the concentration of isoflurane. The chromatographic column was DB-WAX (30 m*530 μm*1 μm). The detector is a hydrogen flame ionization detector (FID). The detector temperature was 300°C, and the injection port temperature was 160°C. The carrier gas was nitrogen at 1.5 ml/min, and the split ratio was 1:1. The heating program is as follows: the initial temperature is 60°C and maintained for 2 min; then it is raised to 200°C at the rate of 10°C/min, maintained for 2 min, and then operated for 2 min. The injection volume was 1 ml. The method has been validated to verify the residue, system adaptability, selectivity, precision and accuracy, standard curve, and lower limit of quantification and stability of isoflurane under different conditions. The in-process analysis was performed on the subject samples to confirm the precision and accuracy of the bioanalysis method.

### Pharmacodynamical Observations

The Modified Observer’s Assessment of Alertness/Sedation (MOAA/S, where 0 = unresponsive and 5 = fully awake) was recorded every 1 min until the subjects were fully awake. The fully awake state was defined as consecutive MOAA/S = 5 for at least three times. The eyelash reflex was tested every 30 s from the beginning of drug injection until the eyelash reflex fully recovered. Central nervous system status (state of consciousness, cognitive function, and mental state) was evaluated after being fully awake. The sedative/anesthesia effects after intravenous EI were evaluated using MOAA/S scores, loss of eyelash reflex, and BIS (Huang et al., 2014). The following items were used to quantitatively assess the efficacy of EI: the minimal MOAA/S score (MOAA/S_min_); area under the delta BIS-time curves (BISAUC_0-t_), in which delta BIS was defined as the change in BIS from baselines to post-dose values; the minimal BIS value (BIS_min_); time to BIS_min_ (t-BIS_min_); time to loss of eyelash reflex; and time to recovery of eyelash reflex.

### Safety Measurements

Subjects were measured by continuous vital signs monitoring (ECG, blood pressure, respiratory rate, heart rate, pulse oxygen saturation, body temperature, and end-tidal carbon dioxide), physical examination, laboratory tests, central nerve system function evaluation (mental status, cognitive function, and consciousness state), injection pain, and adverse effects (focus on coughs based on previous clinical trials). Discomfort reported by subjects or observed by researchers was documented, reviewed, and classified based on the Medical Dictionary for Regulatory Activities (MedDRA, v5.0). Adverse effects were graded as mild, moderate, severe, or life-threatening accordingly.

The trial ceased if the following criteria were met: over half of subjects developed adverse effects that were graded >2, over 1/4 subjects developed grade 3–4 adverse effects, clues indicating that the test drug was intolerable, inappropriate protocols that would fail to evaluate the test drug, or sponsor requiring cessation of the trial.

### Randomization and Statistical Analysis

Statistical analysis was performed with software SAS (version 9.4). Pharmacological parameter analysis was conducted in WinNonlin (version 8.1). Sample size was calculated based on coefficient of variation = 20% (previous trials results), alpha = 0.05, power = 0.8, equivalent margin = 0.8–1.25, and the geometric mean test/reference ratio of NFEI/PFEI within 0.9–1.05. The minimal sample size was 19. C_max_, AUC_0-t_, and AUC_0-∞_ were used to calculate the geometric mean test/reference ratios and CI90 in linear mixed models. Bioequivalence between the new and old formulation of isoflurane was considered when the CI90 of C_max_, AUC_0-t_, and AUC_0-∞_ geometric mean ratio locates within 80%–125%. The other pharmacokinetic parameters of isoflurane were estimated by a non-compartmental model based on the plasma concentration data.

Comparison of pharmacodynamics parameters (t-MOAA/S_min_, BISAUC_0-t_, t-BIS_min_, time to loss of eyelash reflex, and time to eyelash reflex recovery) was conducted using a nonparametric test (Wilcoxon, two-tailed). The incidences of adverse effects were analyzed with the *chi-square* test. The difference was considered significant when *p* < 0.05. Data were presented as mean ± standard variation (SD) where possible.

## Results

### Subjects Demography

**FIGURE 1:**
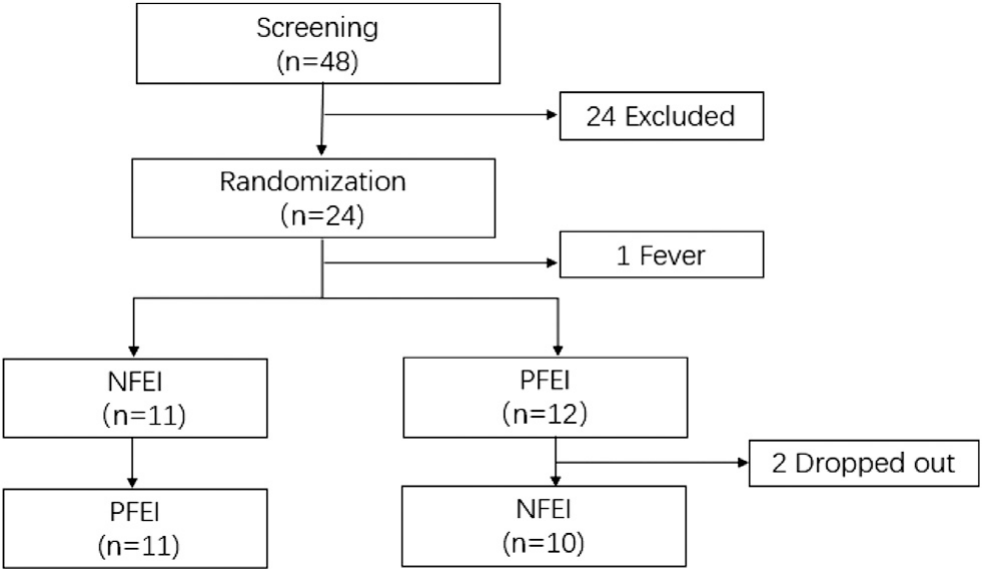
The consort flow diagram for the patient progress through this trial. 48 subjects were enrolled; 24 met the exclusion criteria. The remaining 24 subjects received randomization; one subject had fever before receiving any drug. 11 subjects received new formulation of emulsified isoflurane (NFEI); the other 12 subjects received previous formulation of emulsified isoflurane (PFEI). During the 72-h washout period, the NFEI group had two subjects dropped out, and the remaining 21 subjects completed the whole trial.

The study was performed from October 10th, 2019 to December 6th, 2019. During screening, 24 subjects were excluded. During the crossover, two subjects quitted, and one subject had fever before drug injection. In total, 24 healthy volunteers were enrolled, 23 of them received drug injection, and 21 subjects completed the whole study (Figure 1). There is no statistical difference of demographic characteristics among the subjects (Table 1).

**TABLE 1:** Demographic data.

Demographic information	Value
Age (years)	26.3 ± 4.5
Body weight (kg)	60.3 ± 7.5
BMI (kg/m^2^)	22.4 ± 1.6
Gender, n (%)	
Male	13 (56.5)
Female	10 (43.5)
Modified Mallampati, n (%)	
Grade 1	23 (100.0)

### Pharmacokinetic Properties

**FIGURE 2:**
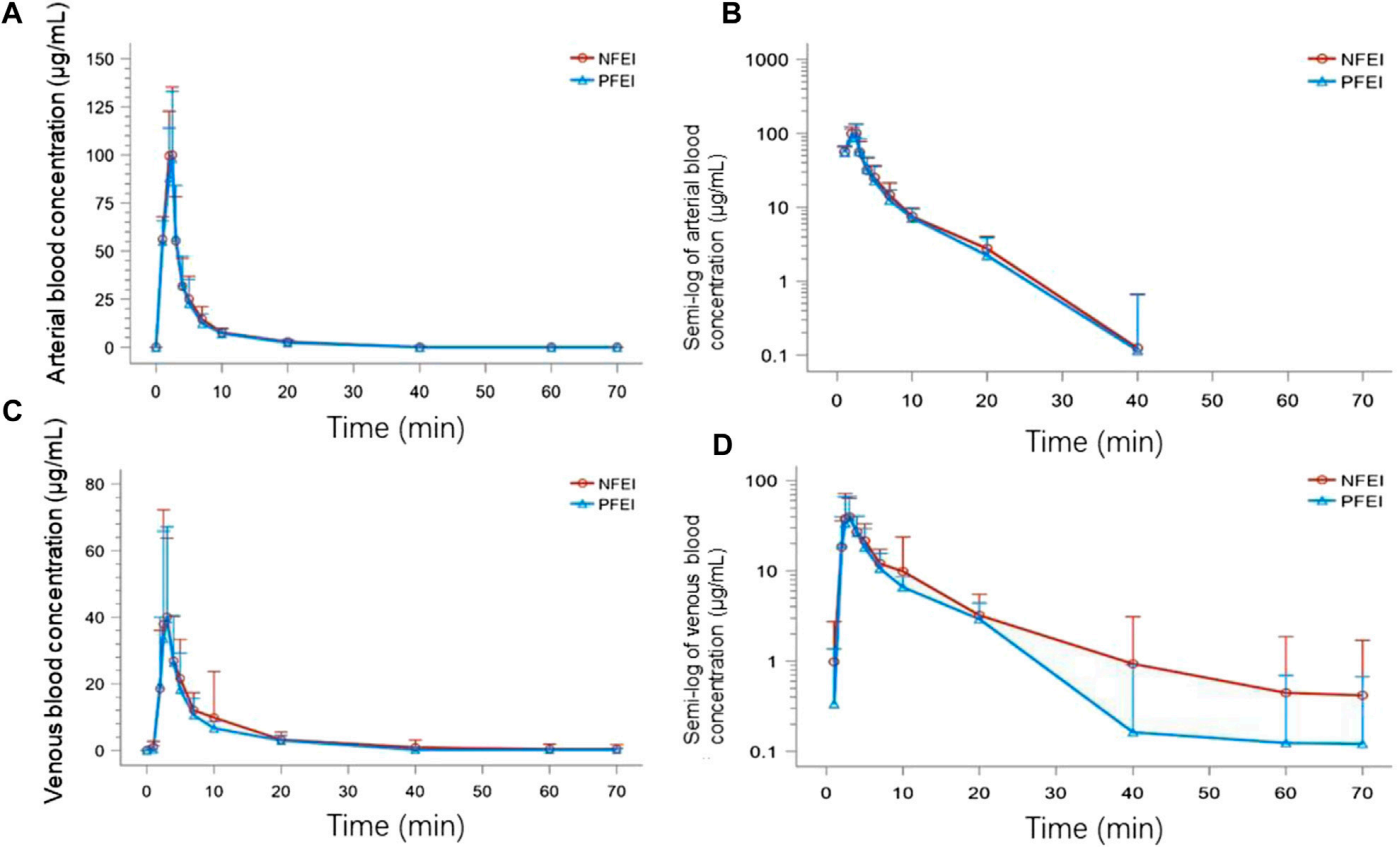
The time–concentration curves of subjects receiving venous injection of 30 mg/kg of new formulation of emulsified isoflurane (NFEI) or previous formulation of emulsified isoflurane (PFEI). The actual arterial **(A)** and venous **(C)** blood isoflurane concentration over time, and the semi-log of arterial **(B)** and venous **(D)** blood drug concentration over time.

There was no clinically important or statistically significant difference of pharmacokinetic parameters between NFEI and PFEI, calculated from arterial or venous blood samples. However, arterial C_max_, AUC_0-t_, and AUC_0-∞_ are higher than the venous ones (Table 2; Figure 2). The intraindividual coefficients of variation for arterial C_max_, AUC_0-t_, and AUC_0-∞_ were 21.7%, 24.8%, and 23.0%, respectively, lower than those for venous values (64.7%, 41.7%, and 31.2%, respectively).

**TABLE 2:** Pharmacokinetic parameters calculated from arterial blood samples after the intravenous new formulation of emulsified isoflurane (NFEI) or previous formulation of emulsified isoflurane (PFEI) in healthy volunteers.

Parameters	NFEI	PFEI
C_max_ (μg/ml)	109.01 ± 29.52	103.39 ± 29.51
AUC_0-t_ (min.μg/ml)	391.77 ± 111.35	362.02 ± 123.79
AUC_0-∞_ (min.μg/ml)	419.14 ± 111.73	391.35 ± 123.96
T_max_ (min)	2.30 ± 0.25	2.37 ± 0.37
t_1/2z_ (min)	5.97 ± 2.02	6.00 ± 2.70
V_z_ (ml/kg)	665.78 ± 288.03	705.11 ± 322.19
CL_z_ (ml/min/kg)	77.33 ± 23.23	86.20 ± 34.19
λ_z_ (min^−1^)	0.14 ± 0.08	0.14 ± 0.06
MRT_0-t_ (min)	3.64 ± 1.02	3.31 ± 1.13
MRT_0-∞_ (min)	5.31 ± 1.60	5.11 ± 1.89
AUC_%Extrap_ (%)	7.00 ± 2.42	8.33 ± 4.16

**TABLE 3:** Pharmacokinetic parameters calculated from venous blood samples after the intravenous new formulation of emulsified isoflurane (NFEI) or previous formulation of emulsified isoflurane (PFEI) in healthy volunteers.

Parameter	NFEI	PFEI
C_max_ (μg/ml)	52.83 ± 26.53	48.47 ± 30.83
AUC_0-t_ (min*μg/ml)	226.01 ± 106.63	201.47 ± 105.53
AUC_0-∞_(min*μg/ml)	279.22 ± 117.11	246.67 ± 110.02
T_max_ (min)	3.19 ± 0.72	3.17 ± 0.76
t_1/2z_ (min)	11.41 ± 8.06	9.61 ± 5.63
V_z_ (ml/kg)	1756.50 ± 884.66	1934.93 ± 1152.92
CL_z_ (ml/min/kg)	125.74 ± 50.14	146.67 ± 70.03
λ_z_ (min^−1^)	0.09 ± 0.04	0.09 ± 0.06
MRT_0-t_ (min)	7.06 ± 4.15	6.44 ± 3.44
MRT_0-∞_(min)	13.79 ± 9.17	12.48 ± 8.08
AUC_%Extrap_ (%)	20.18 ± 12.15	20.68 ± 12.50

The CI90 of arterial C_max_, AUC_0-t_, and AUC_0-∞_ were 92.81%–117.65%, 94.51%–123.96%, and 93.94%–120.80%, respectively, all within the range of 80%–125% (Table 4). The venous ones were 82.43%–171.46%, 90.13%–148.06%, and 94.14%–138.22%, respectively, exceeding the 80%–125% range (Table 5).

**TABLE 4:** Geometric mean test/reference ratios of arterial C_max_, AUC_0-t_, and AUC_0-∞_ and their 90% confidence interval (CI90) from arterial blood samples.

Parameters	Geometric mean		Reference ratio (%)	CI90 (%)
	NFEI	PFEI		
C_max_	103.73	99.27	104.50	92.81∼117.65
AUC_0-t_ (min·μg/ml)	369.18	341.09	108.23	94.51∼123.96
AUC_0-∞_ (min·μg/ml)	396.70	372.39	106.53	93.94∼120.80

**TABLE 5:** Geometric mean test/reference ratios of venous C_max_, AUC_0-t_, and AUC_0-∞_ and their 90% confidence interval (CI90) from venous blood samples.

Parameters	Geometric mean		Reference ratio (%)	CI_90_ (%)
	NFEI	PFEI		
C_max_	45.57	38.34	118.88	82.43∼171.46
AUC_0-t_ (min·μg/ml)	207.27	179.43	115.52	90.13∼148.06
AUC_0-∞_ (min·μg/ml)	262.32	229.96	114.07	94.14∼138.22

### Pharmacodynamical Properties

**FIGURE 3:**
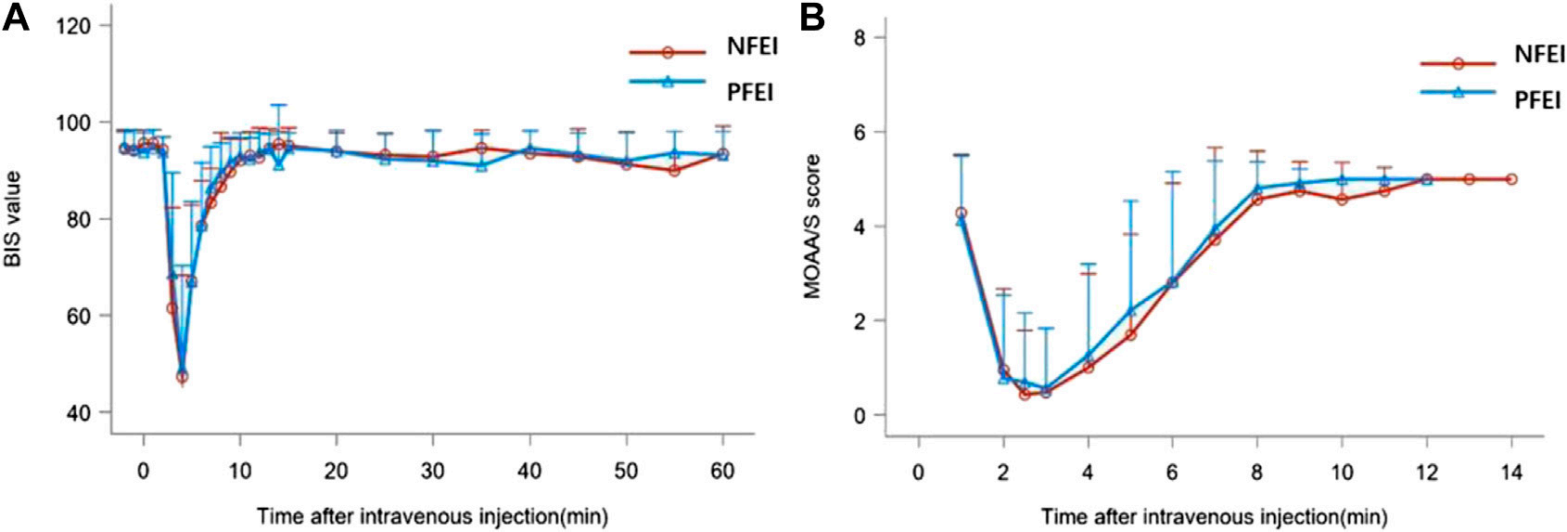
BIS value **(A)** and MOAA/s score **(B)** after intravenous injection of 30 mg/kg of the new formulation of emulsified isoflurane (NFEI) or the previous formulation of emulsified isoflurane (PFEI).

There was no statistically significant difference among the t-BIS_min_, t-MOAA/S_min_, and the time of loss/recovery of eyelash reflex between NFEI and PFEI (Table 6; Figure 3). The time-course of MOAA/S and BIS value between NFEI and PFEI were also similar (Figure 3).

**TABLE 6:** The t-BIS_min_, t-MOAA/S_min_, and eyelash reflection loss/recovery time for PEFI and NEFI.

Parameter	NFEI (N = 21)	PFEI (N = 23)	Statistics	*p* value
*t*-BIS_min_	6.38 ± 11.19	5.78 ± 6.71	−10.500	0.254
*t*-MOAA/S_min_	2.05 ± 0.42	2.07 ± 0.38	−0.500	1.000
Time of eyelash reflex loss	1.79 ± 0.38	1.79 ± 0.30	−2.500	1.000
Time of eyelash reflex recovery	5.87 ± 1.60	5.82 ± 1.33	−3.500	0.839

### Safety Evaluation

There were no serious adverse events, nor suspected unexpected serious adverse reaction observed. Mild vital sign fluctuation was documented in 18 subjects, including tachycardia, hypotension, hypertension, apnea, fever, and peaked T wave (Table 7). Other adverse events were observed: injection pain, swelling of injection site, creatinine elevation, APTT prolongation, PT prolongation, conjugated bilirubin elevation, leukocytes in urine, urine sediment, bacteriuria, triglycerides elevation, upper airway obstruction, dizziness, upper airway infection, cough, and vomiting (Table 8). The incidence for the above adverse events was similar between the two formulations (*p* < 0.05). Some of the adverse events require emergent treatment, such as upper airway obstruction, hypotension, and apnea, but all subjects relieved shortly spontaneously or after treatment. Total incidence of treatment emergent adverse events (TEAEs) for NFEI was 66.7% (14 out of 21 cases), compared with 65.2% (15 out of 23 cases) for PFEI (Table 9), with no statistical differences. The severity of all TEAEs was below grade 2. All TEAEs have spontaneously recovered or relieved.

**TABLE 7:** Change in vital signs after intravenous 30 mg/kg NFEI or PFEI in healthy volunteers.

	NFEI		PFEI	
	n (%)	Case	n (%)	Case
Tachycardia	0	0	1 (4.3)	1
Hypotension	4 (19.0)	4	3 (13.0)	3
Hypertension	1 (4.8)	1	0	0
Apnea	3 (14.3)	3	4 (17.4)	4
Fever	0	0	1 (4.3)	1
Peaked T wave	0	0	1 (4.3)	1

**TABLE 8:** Other adverse events after intravenous injection of 30 mg/kg NFEI or PFEI in healthy volunteers.

Adverse events	NFEI (N = 21)		PFEI (N = 23)	
	n (%)	Case	n (%)	Case
Injection pain	8 (38.1)	8	8 (34.8)	8
Swelling of injection site	1 (4.8)	1	1 (4.3)	1
Creatinine elevation	2 (9.5)	2	1 (4.3)	1
APTT prolongation	2 (9.5)	2	0 (0)	0
PT prolongation	2 (9.5)	2	0 (0)	0
Conjugated bilirubin elevation	1 (4.8)	1	0 (0)	0
Leukocytes in urine	0 (0)	0	1 (4.3)	1
Urine sediment	0 (0)	0	1 (4.3)	1
Bacteriuria	0 (0)	0	1 (4.3)	1
Triglycerides elevation	1 (4.8)	1	0 (0)	0
Upper airway obstruction	3 (14.3)	3	3 (13.0)	3
Dizziness	1 (4.8)	1	0 (0)	0
Upper airway infection	1 (4.8)	1	1 (4.3)	1
Coughing	0 (0)	0	1 (4.3)	1
Vomiting	0 (0)	0	1 (4.3)	1
Total	14 (66.7)	33	15 (65.2)	30

**TABLE 9:** Treatment-emergent adverse events incidence analysis (SS).

	NFEI, n (%)	PFEI, n (%)	Total, n (%)	p value
Adverse events	14 (66.7)	15 (65.2)	18 (78.3)	0.919
Adverse reaction	14 (66.7)	15 (65.2)	18 (78.3)	0.919

## Discussion

The CI90 of arterial C_max_, AUC_0-t_, and AUC_0-∞_ were within the range of 80%–125%. The pharmacodynamical parameters, such as time of loss/recovery of eyelash reflex, time-course of MOAA/S, and BIS value, were similar between NFEI and PFEI. The type and the incidence of adverse events were also similar, with no statistically significant difference.

The pharmacokinetic parameters for NFEI and PFEI were similar. The primary outcomes, CI90 of geometric means for three important parameters, C_max_, AUC_0-t_, and AUC_0-∞_, were between 80% and 125%. This result confirmed our hypothesis that the change of formulation for emulsion does not affect the basic chemo-physical, pharmacokinetic properties of isoflurane, especially drug exposure characteristics.

However, the arterial C_max_, AUC_0-t_, and AUC_0-∞_ were different from the venous values. We chose arterial C_max_, AUC_0-t_, and AUC_0-∞_ for bioequivalence analysis because the venous values from venous blood samples have greater intraindividual coefficient of variation. The reasons for the variation in venous parameters might be as follows: 1) venous blood was drawn from the same arms that had noninvasive blood pressure continuously monitored. The inflation and deflation of the cuff might affect the venous blood returning, therefore leading to the variation of blood concentration. 2) Drawing blood samples from arteries was easier than that from veins; thus venous sample was obtained a little bit slower than arterial ones. Considering that isoflurane was rapidly eliminated in the pulmonary circulation, the slight lag time between might lead to variation in plasma concentration.

The pharmacodynamical properties for NFEI and PFEI were generally similar. Both drugs produced the loss of eyelash reflex within 2 min. The MOAA/S reached the minimal value shortly after the loss of eyelash reflex, approximately 2 min after drug injection. BIS decreased to the minimal value about 6 min after drug application. The development of unconsciousness, unresponsiveness, and depression of electroencephalogram conforms with clinical practice and previous study (Huang et al., 2014). The recovery of eyelash reflex, and the return of MOAA/S and BIS value were rapid and complete; within 10 min, all subjects were fully awake. In addition, the degree of decrease in MOAA/S and BIS values was synchronic between NFEI and PFEI. NFEI mostly inherited the effectiveness of PFEI, in terms of onset, duration, and magnitude.

The safety profile of NFEI did not differ from that of PFEI. No serious adverse effect was observed, indicating that NFEI is equally safe compared with PFEI at this dose. The most common adverse events for both NFEI and PFEI were injection pain, hypotension, apnea, and upper airway obstruction. Injection pain is common in general anesthetics with emulsion as solvent, such as propofol and etomidate. Dose-related hypotension, apnea, and upper airway obstruction are also common for anesthetics. Fortunately, injection pain is transient and spontaneously recovered. Hypotension could be easily treated with fluid infusion or vasoconstrictors; apnea and upper airway obstruction can be easily relieved by the “chin-lift” maneuver in combination with mask ventilation in general anesthesia induction period. There was no cough for NFEI, while one subject experienced cough using PFEI. Although this difference was of no statistical significance confining to the relatively small sample size, NFEI containing smaller particles might have advantage in reducing the risk of cough during general anesthesia induction. Overall, the incidence of adverse events from NFEI was similar to that of those from PFEI.

The incidences of injection pain and cough were less than those in the previous study (Hu et al., 2013; Liu et al., 2013; Wang et al., 2013; Xu et al., 2013; Yang et al., 2013; Huang et al., 2014). One possible explanation was that in a previous study, EI was given in bolus injection within seconds, while in this study, EI was given *via* an infusion pump within 2 min. Another explanation could be that NFEI has a smaller particle size, thus less likely to block pulmonary capillaries.

As the results demonstrated, NFEI has similar pharmacokinetics, pharmacodynamics, and safety properties to PFEI in healthy volunteers at a dose of 30 mg/kg.

## Conclusion

The CI90 of C_max_, AUC_0-t_, and AUC_0-∞_ were 92.81%∼117.65%, 94.51%∼123.96%, and 93.94%∼120.80%, respectively, within the range of 80%–125%, suggesting bioequivalence between the new and previous formulation of emulsified isoflurane.

## Data Availability

The original contributions presented in the study are included in the article/Supplementary Material; further inquiries can be directed to the corresponding author.
